# Quaternized Polysulfone Cross-Linked *N*,*N*-Dimethyl Chitosan-Based Anion-Conducting Membranes

**DOI:** 10.3390/polym11030512

**Published:** 2019-03-18

**Authors:** Gautam Das, Chae Yeon Kim, Dong Ho Kang, Bo Hyeon Kim, Hyon Hee Yoon

**Affiliations:** Department of Chemical and Biological Engineering, Gachon University, Gyeonggi-Do 461-701, Korea; gautam2706@gmail.com (G.D.); kcy9021@gmail.com (C.Y.K.); kdh459@naver.com (D.H.K.); kbh1610@naver.com (B.H.K.)

**Keywords:** anion conducting membrane, composite, chitosan, urea fuel cell

## Abstract

Anion-conducting membranes were obtained following the cross-linking of 1,4-diazoniabicycle[2.2.2]octane functionalized-polysulfone with *N*,*N*-dimethyl chitosan (DMC). The ionic conductivity of the composite membranes was controlled by the amount of DMC. The influence of the amount of DMC on water uptake, swelling ratio, and ionic conductivity of the obtained membrane was studied. The membrane with 2 wt% DMC exhibited an ionic conductivity of 54 mS/cm and 94 mS/cm at 25 °C and 70 °C, respectively. The membrane showed good dimensional stability under hydrated conditions. A urea/O_2_ fuel cell, built using the composite membrane, exhibited a peak power density of 4.4 mW/cm^2^ with a current density of 16.22 mA/cm^2^ at 70 °C.

## 1. Introduction

Fuel cells requiring anion exchange membranes (AEMs) have recently become very popular owing to their good performance and cost effectiveness [1]. In general, anion exchange membrane fuel cells (AEMFCs) utilizing AEMs demonstrate advantages over proton exchange membrane fuel cells (PEMFCs), such as lower cost, better oxygen reduction, and better fuel kinetics [1,2]. However, the ionic conductivity and stability of AEMs are not on a par with those of proton exchange membranes (PEMs). Notwithstanding its low ion exchange capacity (IEC), Nafion (a PEM) exhibits high-proton conductivity (0.1 S/cm) [3,4], which originates from the fast mobility of H^+^, ionic clusterization, good retention of water, and continuous ionic channels bestowed by hydrophilic/hydrophobic phase separation. On the other hand, in case of AEMs, the low mobility of OH^−^ (1/4 of H^+^) combined with weaker dissociation (high pK_b_) results in poor ionic conductivity. Thus, there has been a fundamental challenge to design AEMs with ionic conductivity similar with Nafion. Therefore, currently, research is directed toward fabricating faster hydroxyl transport AEMs, with improved stability [5]. As a result, polymers with new structures [6,7], multifunctional side-chained cationic group’s tethered to polymer chains [8], polymer blends [9,10,11], and composite/nanocomposites [12,13,14,15,16] have all been widely investigated. In general, AEMs with an acceptable performance require a balance between fixed-charge concentration, hydration level, and mechanical properties. For faster transportation of anions, morphology has been reported to play a significant role [7,17,18,19,20]. An AEM with phase-separated morphology analogous to PEMs has been reported to exhibit faster ionic mobility [21,22]. In this regard, composite/nanocomposite material can be suitably used in AEMs, primarily owing to the biphasic nature of the materials [14,16,23,24,25,26,27]. Such materials can form micro-phase separated structures conducive for ion transport.

In this context, composites/nanocomposites such as quaternized polysulfone/layer double hydroxide [28], poly[2,2′-m-(phenylene)-5,5′-bibenzimidazole]/graphene-oxide (PBI/GO) [29], ionic-liquid-coated silica in trimethylamine-functionalized poly(2,6-dimethyl-1,4-phenylene-oxide) [30], quaternized polyhedral-oligomeric-silsesquioxanes (QPOSS)/quaternized polysulfone (QPSU) [31], and cellulose/polysulfone [27] have been used as high-performance AEM materials. Fillers/nanofillers are promising owing to their high surface area and good thermo-mechanical properties [12,32,33,34]. Fillers can be selectively functionalized to induce high-ionic content in AEMs, increasing their ionic conduction. These materials have additional benefits such as good mechanical properties and high-thermal stability.

Chitosan is an inexpensive and abundantly available natural material which has good film formation ability, aptitude for facile modification, excellent mechanical strength, and is environmentally benign. Chitosan contains an anime group attached to its backbone, which can be quaternized and directly used in different applications such as biomedical applications [35,36], membrane design [37,38,39], and water purification. Owing to its hydrophilicity, chitosan is structurally immiscible with organic polymers; consequently, phase separation on the micro- or nano-levels is possible. However, a lack of structural bridging of the chitosan moiety with the polymer would negatively affect the conductivity and mechanical stability. Thus, cross-linking by covalent-bond formation is a benign approach for fabrication of high-performance composite/nanocomposite membrane [27]. Wan et al. [40] synthesized an anion conducting membrane by cross-linking and doping chitosan with glutaraldehyde and KOH, respectively. In another publication, Wan et al. [35] reported enhanced conductivity of AEM by quaternization of chitosan and subsequent cross-linking.

Thus, inspired by the aforementioned findings, a composite membrane was fabricated by combining demethylated chitosan with quaternized polysulfone. The two structures were then cross-linked by a flexible spacer. We expect that cross-linking could simultaneously lead to suppressed swelling and high conductivity as a result of high IEC and distinguished hydrophilic/hydrophobic micro-phase separation.

## 2. Materials and Methods

### 2.1. Materials

Poly(ether sulfone) (M_n_ = 16,000), 1,4-diazabicyclo[2.2.2]octane (DABCO), chloromethyl ethyl ether, tin (IV) chloride, formic acid, formaldehyde, 1,4-dibromo butane (DBB), chitosan (CH) (>75% deacetylated), and *N*,*N*-dimethylformamide (DMF) were purchased from Sigma –Aldrich, Gyeonggi-do, Korea. All other reagents used in the present investigation were of reagent grade.

### 2.2. Synthesis of Dimethyl Chitosan

**Scheme 1:**
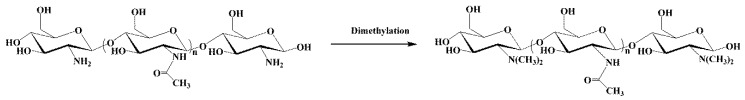
Synthesis of dimethyl chitosan.

The demethylation of chitosan was carried out by a formic acid–formaldehyde methylation (Eschweiler–Clarke) process [41]. Briefly, chitosan (1 g) was mixed with formic acid (3 mL), 37% formaldehyde (4 mL), and 18 mL of deionized (DI) water in a round-bottom flask. The reaction content was allowed to stir for 120 h at 70 °C. The reaction mass was added to 1 mol/L NaOH (pH 12) to obtain a viscous yellowish gel, which was then dissolved in 1 mol/L HCl (pH 4). The gel was then repeatedly washed with water to remove any impurities. Subsequently, the gel was dissolved in DI water at pH 4 (adjusted with 1 M HCl), and then dialyzed against DI water for 3 days (by changing the buffer twice daily). Finally, the product was lyophilized to obtain *N*,*N*-dimethyl chitosan (DMC) (Scheme 1).

### 2.3. Chloromethylation and Quaternization of Polysulfone

Polysulfone (PSf) was chloromethylated using a method similar to the one reported by Zhang et al. [27,42]. Briefly, in 80 mL of chloroform, 2 g of PSf was dissolved at room temperature, and to this solution 120 µL of SnCl_4_ (anhydrous) and 3.8 mL of cholomethylethyl ether was added dropwise. The reaction was allowed to proceed for 24 h at 40–45 °C under N_2_ purging. Afterwards, the reaction mixture was precipitated in methanol and stirred overnight. The precipitate was washed with methanol and dried in a vacuum oven overnight. The dried product was denoted as ClPSf (chloromethylated PSf).

The quaternization reaction was carried out first by dissolving ClPSf (2 g) in DMF (20 mL), and then adding DABCO (1.2 g) in a three-necked round-bottom flask under N_2_ atmosphere. After continuous stirring for 24 h at 70 °C, the content of the flask was precipitated in diethyl ether. The precipitate was repeatedly washed with diethyl ether and ethyl acetate and dried in a vacuum oven overnight to obtain quaternized PSf (QPSf).

### 2.4. Cross-Linking and Membrane Fabrication

**Scheme 2:**
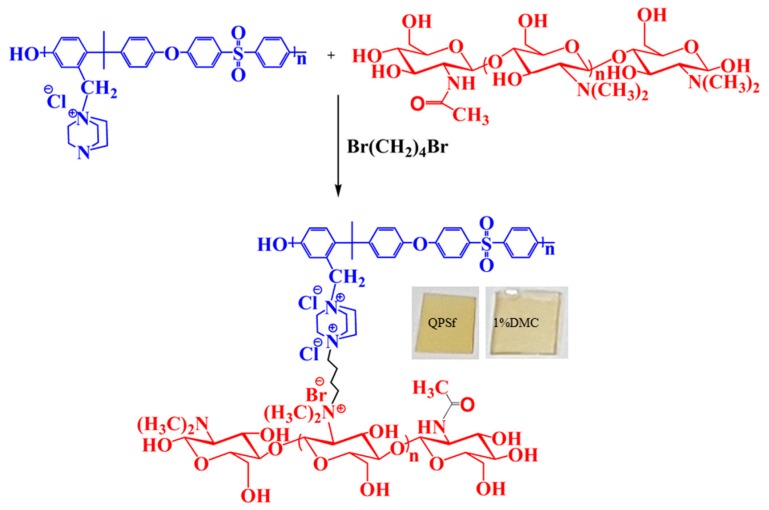
Composite membrane fabrication.

The composite membrane was fabricated as shown in Scheme 2. The aminated moieties in QPSF and DMC were cross-linked with 1,4-dibromobutane as described in earlier published reports [27,43,44]. In a typical procedure, QPSf was first dissolved in DMF (20 wt% solution). To the above solution, DMC (0, 1, 2, and 3 wt%) dispersed in DMF (2 mg/mL) was added under mechanical stirring at 70 °C for 24 h. Next, pre-gelation was performed by adding 1,4-dibromobutane for 30 min. The content was then poured into a glass Petri dish and dried at 70 °C for 24 h in a vacuum oven to obtain the cross-linked membrane. The dried membranes were then weighed to a constant weight and were coded as QPSf (without cross-linking), QPSf/DBB (cross-linked with DBB), QPSfDMC1, QPSfDMC2, and QPSfDMC3, where the digit indicates the weight percent of DMC.

### 2.5. Water Uptake and Swelling Ratio

The water uptake (WU) and the swelling ratio (SR) in-plane (*SR_ip_*) and through-plane (*SR_tp_*) of the membranes were measured by first taking the dry weight (*W_dry_*) and then immersing the membranes in DI water for 24 h. The membranes were then taken out and carefully wiped to remove excess surface water, and subsequently, the weight (*W_wet_*) was determined. The swelling ratio *SR_ip_* and *SR_tp_* were determined before and after immersion by measuring the length (cm) and thickness (cm) of the membranes, respectively. Both WU and SR were then calculated using Equations (1)–(3):

(1) $$WU=\;\frac{W_{wet}-W_{dry}}{W_{dry}}$$

(2) $$SR_{ip}=\frac{L_{wet}-L_{dry}}{L_{dry}}$$

(3) $$SR_{tp}=\frac{T_{wet}-T_{dry}}{T_{dry}}$$

In the above equations, the thicknesses in the wet and dry state are *T_wet_* and *T_dry_*, respectively, whereas the lengths in the wet and dry states are denoted by *L_wet_*, and *L_dry_*, respectively.

### 2.6. Ion Exchange Capacity (IEC)

Membranes of specific sizes were cut and immersed in a 100 mmol/L NaOH aqueous solution for 24 h. The OH^-^ exchanged membranes were then equilibrated with DI water, and then the membranes were immersed in 20 mL of a 10 mmol/L HCl aqueous solutions for 48 h. The ion-exchange capacity (IEC; mmol/g) of the membrane was then calculated as:

(4) $$IEC=\frac{\left(V_{b}-V_{r}\right)\times C_{NaOH}}{W_{dry}}$$

where, *V_b_* and *V_r_* denote the volume consumed during titration for the blank solution with the samples, respectively. *C_NaOH_* represent the concentration of NaOH and *W_dry_* is the weight of the dried membranes.

### 2.7. Analysis/Characterization

A JASCO FTIR 300E Fourier transform infrared (FTIR, Easton, MD, USA) spectrometer was used to record the attenuated total reflectance FTIR (ATR-FTIR) spectra of all of the samples. The ^1^H-NMR spectra of PSf, ClPSf, and QPSf were recorded using an A 400 MHz Fourier transform nuclear magnetic resonance (FT-NMR) spectrometer (Avance II, Bruker Biospin, Billerica, MA, USA). The morphologies of chitosan, DMC, and the cross-sectional images of the composites were analyzed using a scanning electron microscope (SEM) (Hitachi S-4700, Tokyo, Japan). The membrane samples were dipped in liquid N_2_ and were broken; the broken pieces were then sputtered with platinum for imaging.

### 2.8. Ionic Conductivity

A Solartron 1255B and 1287 (Solartron Analytical, Farnborough, UK) frequency response analyzer was used to obtain the Nyquist plot of the exchange membranes. A two-probe conductivity cell was used for these measurements. An inert atmosphere was maintained during the impedance measurement. The Nyquist plots were obtained at amplitude of 50 mV, for the frequencies in the range of 100 kHz to 7 MHz. The ionic conductivity was then determined as:

(5) $$\sigma =\frac{l}{\left(R\times A\right)}$$

where *l* (cm) is the separation between the two electrodes; *R* (ohm) denotes the resistance obtained from the Nyquist plot, and *A* (cm^2^) represents the surface area.

### 2.9. Fuel Cell Testing and Analysis

Current versus voltage (IV) curves for urea fuel/O_2_ fuel cell were obtained using a potentiostat–galvanostat (Biologic SP-240, Seyssinet-Pariset, France) connected to a current booster (4 A) controlled by the EC-lab 11.01 software for automated data collection. The urea/O_2_ fuel cell consisted of a flow of an alkaline urea solution of 0.33 mol/L at the anode side and moist oxygen at the cathode side. The anode and cathode catalysts were Ni/C (30%) and PtRu/C (Fuel Cell Store, Texas, USA), respectively. The loading of Ni/C and PtRu/C were kept at 5 mg/cm^2^ and 1.5 mg/cm^2^, respectively. The active area of the unit cell was 5 cm^2^; the urea flow was maintained at a rate of 10 mL min^−1^ using a peristaltic pump. At the cathode, humidified oxygen (100 mL min^−1^) was used as the electron acceptor.

## 3. Results and Discussions

### 3.1. FTIR and NMR Analysis

**Figure 1:**
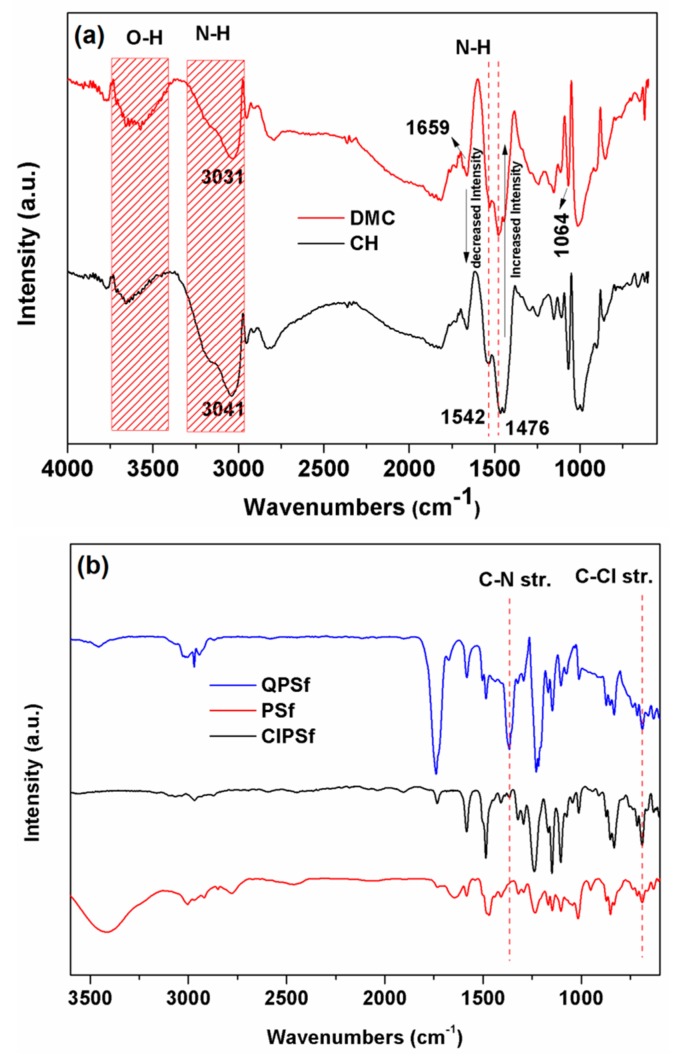
Fourier transform infrared (FTIR) spectra (**a**) chitosan (CH) and *N*,*N*-dimethyl chitosan (DMC); and (**b**) polysulfone (PSf), chloromethylated polysulfone (ClPSf) and quaternized PSf/dibromo butane (QPSf/DBB).

The FTIR spectra of DMC and chitosan (CH) are shown in Figure 1a. In the FTIR spectra, the band at 3031–3041 cm^−1^ originates from the N–H stretching vibrations. Further, the characteristics absorption bands of chitosan appeared at ~1659 cm^−1^ owing to the amide stretching and ~1542 cm^−1^ owing to the N–H bending vibration. Upon methylation, the relative intensity of the band at ~1542 cm^−1^ corresponding to the N–H bending vibration and N–H stretching band at 3031 cm^−1^ for DMC [37,41] decreased relative to that of amide stretching band at 1659 cm^−1^, similar to reports published elsewhere [37,41]. Moreover, the band at ~1476 cm^−1^ was attributed to the C–H, of the –N(CH_3_)_2_ group, and its intensity increased after the formation of *N*,*N*-dimethyl chitosan, indicating the substitution of NH by –CH_3_ groups [45]. Thus, the FTIR spectra confirmed the successful methylation reaction.

The FTIR spectra of the modified polysulfone are shown in Figure 1b, and the appearance of a C–Cl stretching vibration band at ~685 cm^−1^ indicates the chloromethylation of the PSf [27,46]. The substitution of the –Cl group by DABCO was evident in the QPSf by observations of the diminished intensity of the C–Cl stretching band. In addition, the appearance of the C–N stretching frequency at around 1366 cm^−1^ in the spectrum of the QPSf indicated the attachment of DABCO to the PSf backbone reactions [46].

### 3.2. NMR Analysis

**Figure 2:**
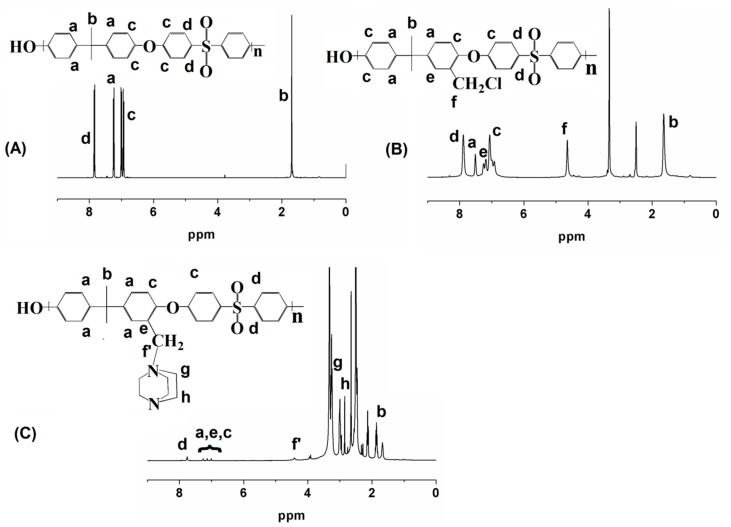
**^1^**H-NMR spectra (**A**) PSf, (**B**) ClPSf, and (**C**) QPSf.

The ^1^H-NMR spectra of the PSf, ClPSf, and QPSf are shown in Figure 2. As can be seen, a signal originating at 4.64 ppm in the ClPSf from the proton of the –CH_2_Cl group indicates a successful chloromethylation reaction [27]. This peak diminishes and the appearance of a small peak at ~4.40 ppm with an up-field shift confirms the quaternization of the ClPSf. Furthermore, in the QPSf spectrum, observation of characteristic peak of DABCO at ~3.0 ppm affirms the attachment of DABCO to the PSf structure [19,27]. The degree of substitution (DS) was calculated using the following equation;

(6) $$DS=[\left(2A\left(H_{f}\right)/A\left(H_{d}\right)\right]\times 100\%$$

where, AH_f_ and AH_d_ are the areas of H_f_ (proton in the −CH_2_Cl) and H_d_ (the proton adjacent to the −SO_2_− group in the aromatic ring) in the spectra of the ClPSf. The DS was found to be 133% after 12 h of the chloromethylation reaction.

### 3.3. SEM Analysis

**Figure 3:**
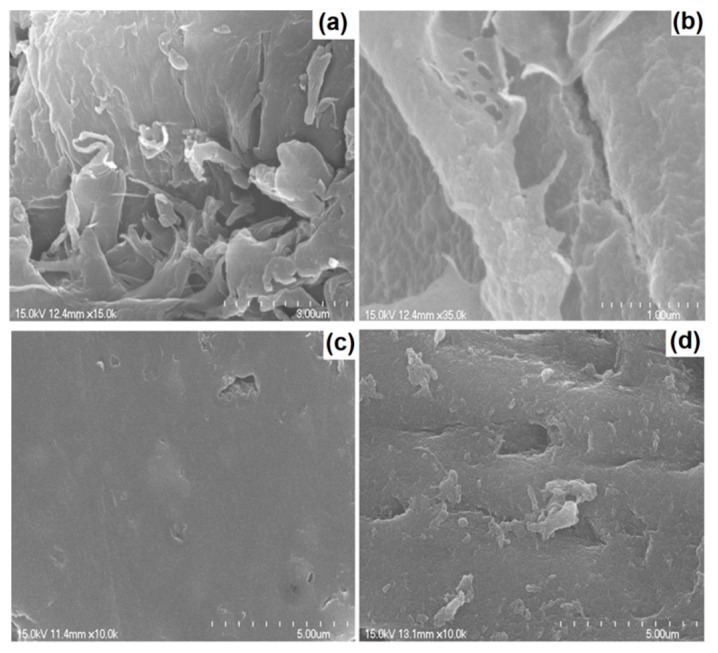
SEM micrographs of (**a**) chitosan, (**b**) DMC, (**c**) QPSf/DBB, and (**d**) quaternized PSf composited with 2 wt% *N*,*N*-dimethyl chitosan (QPSfDMC2).

The SEM micrographs are shown in Figure 3. As can be observed, chitosan was seemingly fibrous in nature. Interestingly, the subsequent quaternization reaction did not change the morphology of the fibers significantly (Figure 3a,b). The morphology of the membranes were observed by taking a cross-sectional area as shown in Figure 3c,d. The cross-sectional image of the QPSf/DBB membrane showed a uniform and smooth surface with no visible macroscopic phase segregation. On the other hand, the QPSfDMC2 exhibited a rough and connective surface. This might be due to the cross-linking reaction which connects the components through a chemical bond, indicating that the composite is compact and homogeneous.

### 3.4. Properties of the Composite Membranes

**Figure 4:**
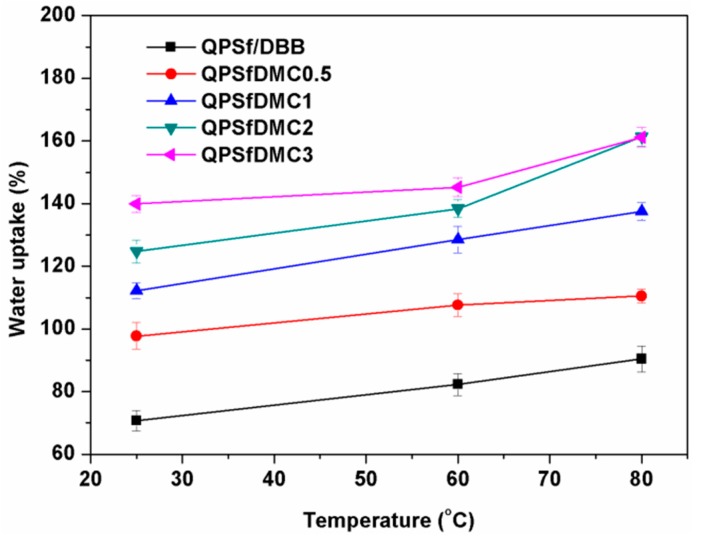
Water uptake with temperature for the different membranes.

The IEC, water uptake, and swelling ratio of the composite membranes are listed in Table 1. The presence of the fixed-charge concentration is evident from the IEC. The IEC value increased upon the formation of the composites; pristine QPSf/DBB showed an IEC of 1.20 mmol/g, which increased to 1.68–2.34 mmol/g for the composite membranes. Thus, it is clear that demethylation and subsequent cross-linking of DMC with the QPSf matrix increased the overall charge density in the AEMs. In general, with an increase in the IEC, the water uptake of the AEM increases. Thus, the water uptakes were measured for each membrane and compared (Table 1). The pristine QPSf/DBB membrane exhibited a water uptake of 70.72%; this value was seen to increase substantially upon the fabrication of the DMC/QPSf composite membranes. Table 1 depicts the water uptake for all of the membranes. The water uptake of the composite membranes at room temperature were found to be in the 97.75–139.89% range. Increasing the temperature further enhanced the water uptake of all the membranes (Figure 4). As mentioned above the increase in the water uptake was primarily owing to the increase in the IEC value. Moreover, since chitosan is hydrophilic in nature owing to the oxygenated functionalities, the increase in the water uptake of the composite membranes can also be attributed to these polar functional groups. The water uptake plays an important role in ionic mobility; therefore, it can be presumed that higher water uptake will result in better ionic conductivity. Thus, it can be concluded that increasing the IEC of the AEMs positively contributed to higher WU in the composite AEMs.

However, excessive water uptake might result in the mechanical instability of the membranes. Thus, the through-plane and in-plane swelling ratios were estimated for determining the stability of the membranes. The swelling ratio for both SR_ip_ and SR_tp_ were found to be in the 22.71–33.51% range (Table 1). Despite the higher swelling, the membranes displayed good dimensional integrity and the membranes remained mechanically robust. This might be due to the cross-linking reaction. The cross-linking reaction between DMC and QPSf restricts chain-drifting upon swelling, thereby preventing the membranes from disintegrating.

**Figure 5:**
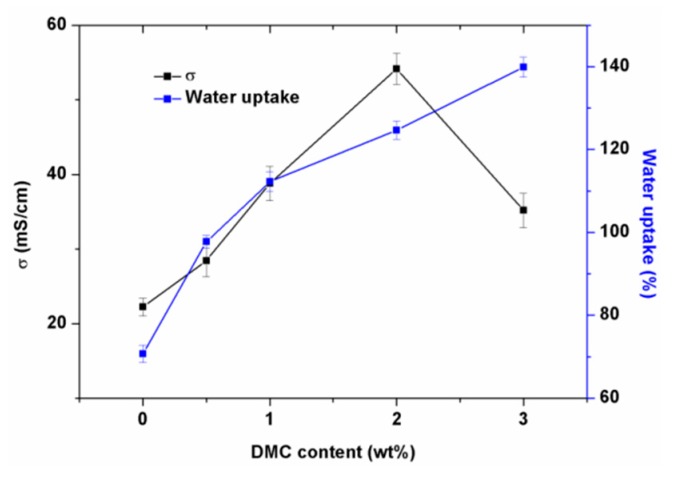
Ionic conductivity and water uptake versus the DMC content measured at room temperature.

**Figure 6:**
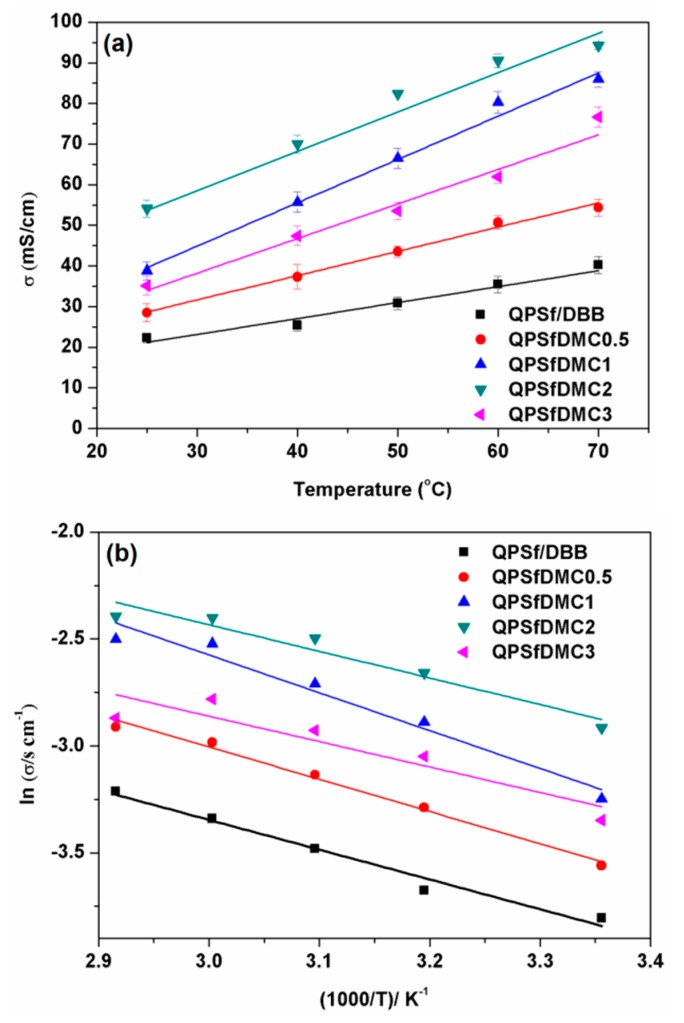
(**a**) Ionic conductivity versus temperature and (**b**) the Arrhenius plot for the different membranes.

The ionic conductivities of the composites membranes are presented in Table 1. To avoid carbonate contamination, the membranes were stored in the N_2_ atmosphere in a sealed container, and during conductivity measurements N_2_ was continuously flushed into the conductivity cell. The ionic conductivity of pristine QPSf/DBB was found to be ~22.25 mS/cm at 25 °C, and the conductivity increased for the composite membranes. In the case of QPSfDMC0.5, the ionic conductivity increased ~1.3-fold compared with the pristine membrane, whereas QPSfDMC2 exhibited a ~2.5-fold increase compared with the pristine membrane (Table 1). The introduction of two different phases facilitated the formation of a more distinct hydrophilic/hydrophobic phase separation. These micro-phase separation resulted in well-connected ionic channels facilitating faster hydroxyl transport. A correlation plot of water content with ionic conductivity is shown in Figure 5, and as can be seen, the ionic conductivity increases as the water content increases. Water content plays an important role in connecting ionic domains, resulting in continuous pathways [3,46]. It has been reported that ionic clusterization is necessary for better connection of ionic domains [3,4]. However, higher water content might dilute the carrier charges and decrease ionic conductivity as was the case of QPSfDMC3 [46]. The variation of the ionic conductivity with temperature was also studied, for temperature in the 25–70 °C range. The conductivity of the composite membranes was higher than that of pristine QPSf/DBB for all temperatures in the studied range. The QPSf/DBB displayed an ionic conductivity of ~40 mS/cm at 70 °C, whereas a maximal ionic conductivity of ~94 mS/cm was achieved for QPSfDMC2 at 70 °C (Figure 6a). This indicates that hydroxyl ions can transverse swiftly by efficiently utilizing hydrated ionic channels. Thus, the quaternized PSf which exhibits a good compatibility with DMC induced by cross-linking resulted in well-separated ionic domains. On the other hand, the ionic conductivity was reduced for QPSfDMC3; higher content of chitosan might exhibit a “blocking effect” that restricts free ion transport, thus decreasing the conductivity [47]. The activation energy of the membranes was in the 9.88–14.71 kJ/mol range, as determined from the slope of the conductivity versus the 1/T plot (Figure 6b). This activation energy indicates that the composite membranes offered less resistance to the hydroxyl ion movement.

### 3.5. Fuel Cell Analysis

**Figure 7:**
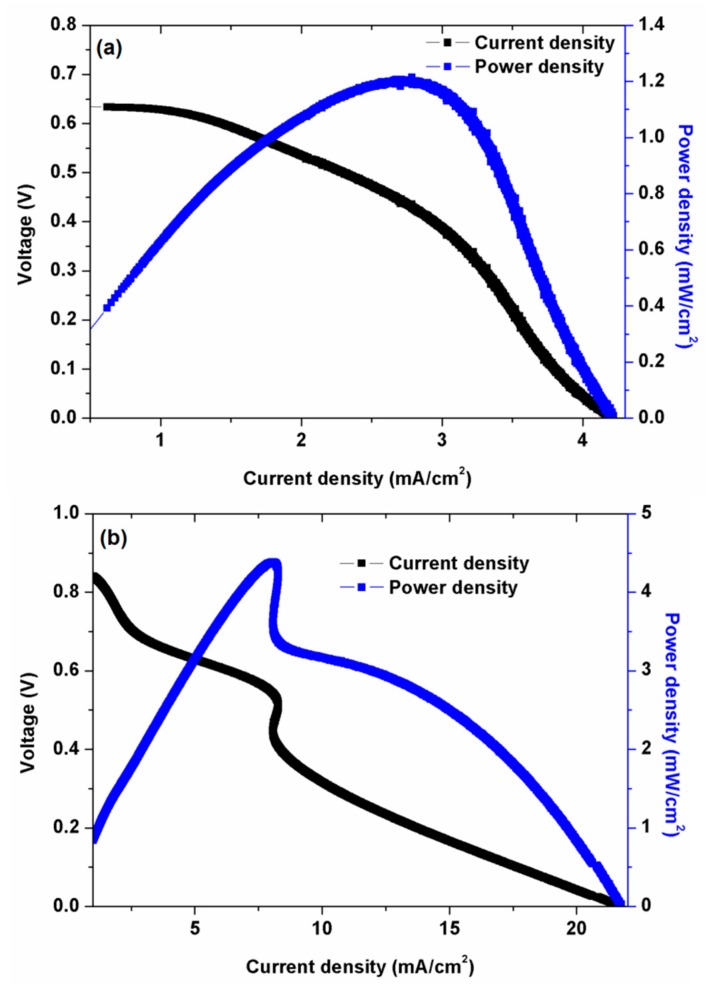
Urea/O_2_ test results for (**a**) QPSf/DBB and (**b**) QPSfDMC2 at 70 °C. The anolyte was 0.33 mol/L urea + 1 mol/L of KOH, while humidified O_2_ was used as the catholyte.

A direct urea fuel cell test was performed using QPSf/DBB and QPSFDMC2 as the membranes. In the case of QPSf/DBB, the unit cell displayed an open circuit voltage (OCV) of 0.63 V at 60 °C (Figure 7a). The peak power density at this temperature was found to be 1.2 mW/cm^2^. On the other hand, when QPSfDMC2 was used as the cell membrane, the cell displayed an OCV of 0.84 V with a peak power density of 4.4 mW/cm^2^ (Figure 7b). It was observed in this case that the QPSfDMC2 membranes performed better compared with previous reports for a urea/O_2_ fuel cell using Ni/C as the anode catalysts [48,49]. The low-cost composite membranes in the current study were feasible and showed potential as AEM in practical applications.

## 4. Conclusions

In the present study, dimethyl chitosan cross-linked polysulfone composite was evaluated as an AEM. Dimethyl chitosan increased the ionic content of polysulfone and induced micro-phase separated morphology, thereby increasing the membrane ionic conductivity. Consequently, a hydroxyl conductivity of 94 mS/cm was achieved for 2 wt% of DMC loading. Further, a urea/O_2_ fuel cell test revealed a power density 4.4 mW/cm^2^ with an OCV of 0.84 V. The membranes were dimensionally stable and exhibited overall good performance as AEMs.

## Figures and Tables

**Table 1:** Properties of the membranes.

Membrane Code	Water Uptake (%), @ RT ^*^	Ion Exchange Capacity (mmol/g)	Through-Plane Swelling Ration (%) @ RT	In-Plane Swelling Ratio (%) @ RT	Ionic Conductivity (mS/cm) @ 25 °C	Gel Fraction (%)
QPSf/DBB	70.72 ± 2.10	1.20 ± 0.14	25.75 ± 2.65	28.10 ± 2.52	22.25 ± 1.22	80
QPSfDMC0.5	97.75 ± 1.57	1.68 ± 0.25	28.21 ± 2.41	31.89 ± 2.43	28.45 ± 2.24	90
QPSfDMC1	112.25 ± 2.31	2.08 ± 0.11	30.33 ± 2.17	33.51 ± 1.55	38.81 ± 2.37	95
QPSfDMC2	124.72 ± 2.20	2.34 ± 0.17	32.31 ± 1.89	30.48 ± 1.85	54.15 ± 2.10	97
QPSfDMC3	139.89 ± 2.41	1.95 ± 0.10	25.07 ± 1.78	22.71 ± 2.17	35.17 ± 2.33	94

* RT = Room temperature.

## References

[1] Varcoe J.R., Slade R.C. (2005). Prospects for Alkaline Anion-Exchange Membranes in Low Temperature Fuel Cells. Fuel Cells.

[2] Varcoe J.R., Atanassov P., Dekel D.R., Herring A.M., Hickner M.A., Kohl P.A., Kucernak A.R., Mustain W.E., Nijmeijer K., Scott K. (2014). Anion-exchange membranes in electrochemical energy systems. Energy Environ. Sci..

[3] Ochi S., Kamishima O., Mizusaki J., Kawamura J. (2009). Investigation of proton diffusion in Nafion^®^117 membrane by electrical conductivity and NMR. Solid State Ionics.

[4] Mauritz K.A., Moore R.B. (2004). State of understanding of Nafion. Chem. Rev..

[5] Pham T.H., Olsson J.S., Jannasch P. (2017). N-Spirocyclic Quaternary Ammonium Ionenes for Anion-Exchange Membranes. J. Am. Chem. Soc..

[6] Zhu L., Zimudzi T.J., Li N., Pan J., Lin B., Hickner M.A. (2016). Crosslinking of comb-shaped polymer anion exchange membranes via thiol–ene click chemistry. Polym Chem..

[7] Weiber E.A., Meis D., Jannasch P. (2015). Anion conducting multiblock poly(arylene ether sulfone)s containing hydrophilic segments densely functionalized with quaternary ammonium groups. Polym Chem..

[8] Han J., Zhu L., Pan J., Zimudzi T.J., Wang Y., Peng Y., Hickner M.A., Zhuang L. (2017). Elastic long-chain multication cross-linked anion exchange membranes. Macromolecules.

[9] Yu Xu P., Yi Guo T., Hui Zhao C., Broadwell I., Gen Zhang Q., Liu Q.L. (2013). Anion exchange membranes based on poly(vinyl alcohol) and quaternized polyethyleneimine for direct methanol fuel cells. J. Appl. Polym. Sci..

[10] Wu L., Xu T. (2008). Improving anion exchange membranes for DMAFCs by inter-crosslinking CPPO/BPPO blends. J. Membr. Sci..

[11] Morandi C.G., Peach R., Krieg H.M., Kerres J. (2015). Novel morpholinium-functionalized anion-exchange PBI-polymer blends. J. Mater. Chem. A.

[12] Zarrin H., Fu J., Jiang G., Yoo S., Lenos J., Fowler M., Chen Z. (2015). Quaternized Graphene Oxide Nanocomposites as Fast Hydroxide Conductors. ACS Nano.

[13] Li J., Yan X., Zhang Y., Zhao B., He G. (2016). Enhanced hydroxide conductivity of imidazolium functionalized polysulfone anion exchange membrane by doping imidazolium surface-functionalized nanocomposites. RSC Adv..

[14] Yang T., Li Z., Lyu H., Zheng J., Liu J., Liu F., Zhang Z., Rao H. (2018). A graphene oxide polymer brush based cross-linked nanocomposite proton exchange membrane for direct methanol fuel cells. RSC Adv..

[15] Yang Q., Lin C.X., Liu F.H., Li L., Zhang Q.G., Zhu A.M., Liu Q.L. (2018). Poly (2,6-dimethyl-1,4-phenylene oxide)/ionic liquid functionalized graphene oxide anion exchange membranes for fuel cells. J. Membrane Sci..

[16] Wang C., Lin B., Qiao G., Wang L., Zhu L., Chu F., Feng T., Yuan N., Ding J. (2016). Polybenzimidazole/ionic liquid functionalized graphene oxide nanocomposite membrane for alkaline anion exchange membrane fuel cells. Mater. Lett..

[17] Zhang H., Shi B., Ding R., Chen H., Wang J., Liu J. (2016). Composite Anion Exchange Membrane from Quaternized Polymer Spheres with Tunable and Enhanced Hydroxide Conduction Property. Ind. Eng. Chem. Res..

[18] Wu X., Chen W., Yan X., He G., Wang J., Zhang Y., Zhu X. (2014). Enhancement of hydroxide conductivity by the di-quaternization strategy for poly(ether ether ketone) based anion exchange membranes. J. Mater. Chem. A.

[19] Wang X., Li M., Golding B.T., Sadeghi M., Cao Y., Yu E.H., Scott K. (2011). A polytetrafluoroethylene-quaternary 1,4-diazabicyclo-[2.2.2]-octane polysulfone composite membrane for alkaline anion exchange membrane fuel cells. Int. J. Hydrog. Energy.

[20] Tsai T.H., Maes A.M., Vandiver M.A., Versek C., Seifert S., Tuominen M., Liberatore M.W., Herring A.M., Coughlin E.B. (2013). Synthesis and structure–conductivity relationship of polystyrene-block-poly (vinyl benzyl trimethylammonium) for alkaline anion exchange membrane fuel cells. J. Polym. Sci. B.

[21] Lee K.H., Cho D.H., Kim Y.M., Moon S.J., Seong J.G., Shin D.W., Sohn J.-Y., Kim J.F., Lee Y.M. (2017). Highly conductive and durable poly(arylene ether sulfone) anion exchange membrane with end-group cross-linking. Energy Environ. Sci..

[22] He Y., Si J., Wu L., Chen S., Zhu Y., Pan J., Ge X., Yang Z., Xu T. (2016). Dual-cation comb-shaped anion exchange membranes: Structure, morphology and properties. J. Membr. Sci..

[23] Nagarale R.K., Gohil G.S., Shahi V.K., Rangarajan R. (2005). Preparation of organic–inorganic composite anion-exchange membranes via aqueous dispersion polymerization and their characterization. J. Colloid Interface Sci..

[24] Liu J., Qu R., Peng P., Liu W., Chen D., Zhang H., Liu X. (2016). Covalently functionalized graphene oxide and quaternized polysulfone nanocomposite membranes for fuel cells. RSC Adv..

[25] Nonjola P.T., Mathe M.K., Modibedi R.M. (2013). Chemical modification of polysulfone: Composite anionic exchange membrane with TiO2 nano-particles. Int. J. Hydrog. Energy.

[26] Liang N., Liu W., Zuo D., Peng P., Qu R., Chen D., Zhang H. (2018). Quaternized polysulfone-based nanocomposite membranes and improved properties by intercalated layered double hydroxide. Polym. Eng. Sci..

[27] Das G., Park B.J., Yoon H.H. (2016). A bionanocomposite based on 1, 4-diazabicyclo-[2.2. 2]-octane cellulose nanofiber cross-linked-quaternary polysulfone as an anion conducting membrane. J. Mater. Chem. A.

[28] Di Vona M.L., Casciola M., Donnadio A., Nocchetti M., Pasquini L., Narducci R., Knauth P. (2017). Anionic conducting composite membranes based on aromatic polymer and layered double hydroxides. Int. J. Hydrog. Energy.

[29] Yu B.-C., Wang Y.-C., Lu H.-C., Lin H.-L., Shih C.-M., Kumar S.R., Lue S.J. (2017). Hydroxide-ion selective electrolytes based on a polybenzimidazole/graphene oxide composite membrane. Energy.

[30] Chen N., Liu Y., Long C., Li R., Wang F., Zhu H. (2017). Enhanced performance of ionic-liquid-coated silica/quaternized poly(2,6-dimethyl-1,4-phenylene oxide) composite membrane for anion exchange membrane fuel cells. Electrochim. Acta.

[31] Miao L., Wang X., Fu Y., Hu B., Bai Y., Lü C. (2017). Quaternized polyhedral oligomeric silsesquioxanes (QPOSS) modified polysulfone-based composite anion exchange membranes. Solid State Ionics.

[32] Potts J.R., Dreyer D.R., Bielawski C.W., Ruoff R.S. (2011). Graphene-based polymer nanocomposites. Polymer.

[33] Yuan Y., Shen C., Chen J., Ren X. (2018). Synthesis and characterization of cross-linked quaternized chitosan/poly (diallyldimethylammonium chloride) blend anion-exchange membranes. Ionics.

[34] Wang J., He R., Che Q. (2011). Anion exchange membranes based on semi-interpenetrating polymer network of quaternized chitosan and polystyrene. J. Colloid Interface Sci..

[35] Wan Y., Peppley B., Creber K.A.M., Bui V.T., Halliop E. (2008). Quaternized-chitosan membranes for possible applications in alkaline fuel cells. J. Power Sources.

[36] Li M., Chen Q., Ma M., Liu X., Dong K., Zhang Y., Lu T. (2019). Electrophoretic deposition of core-shell Ag@MSN incorporated-chitosan coatings with biocompatible and antibacterial activities. Mater. Lett..

[37] Britto D., Assis O.B.G. (2007). A novel method for obtaining a quaternary salt of chitosan. Carbohydr. Polym..

[38] Liao G.-M., Yang C.-C., Hu C.-C., Pai Y.-L., Lue S.J. (2015). Novel quaternized polyvinyl alcohol/quaternized chitosan nano-composite as an effective hydroxide-conducting electrolyte. J. Membr. Sci..

[39] Feketeföldi B., Cermenek B., Spirk C., Schenk A., Grimmer C., Bodner M., Koller M., Ribitsch V., Hacker V. (2016). Chitosan-Based Anion Exchange Membranes for Direct Ethanol Fuel Cells. J. Membr. Sci. Technol..

[40] Wan Y., Peppley B., Creber K.A.M., Bui V.T., Halliop E. (2006). Preliminary evaluation of an alkaline chitosan-based membrane fuel cell. J. Power Sources.

[41] Verheul R.J., Amidi M., van der Wal S., van Riet E., Jiskoot W., Hennink W.E. (2008). Synthesis, characterization and in vitro biological properties of O-methyl free N,N,N-trimethylated chitosan. Biomaterials.

[42] Zhang F., Zhang H., Qu C. (2011). Imidazolium functionalized polysulfone anion exchange membrane for fuel cell application. J. Mater. Chem. A.

[43] Taheri M., Ghiaci M., Shchukarev A. (2018). Cross-linked chitosan with a dicationic ionic liquid as a recyclable biopolymer-supported catalyst for cycloaddition of carbon dioxide with epoxides into cyclic carbonates. New J. Chem..

[44] Chu E., Sidorenko A. (2013). Surface reconstruction by a “grafting through” approach: Polyacrylamide grafted onto chitosan Film. Langmuir.

[45] Wu M., Long Z., Xiao H., Dong C. (2017). Preparation of N, N, N-trimethyl chitosan via a novel approach using dimethyl carbonate. Carbohydr. Polym..

[46] DeCaluwe S.C., Baker A.M., Bhargava P., Fischer J.E., Dura J.A. (2018). Structure-property relationships at Nafion thin-film interfaces: Thickness effects on hydration and anisotropic ion transport. Nano Energy.

[47] Hu B., Miao L., Zhao Y., Lü C. (2017). Azide-assisted crosslinked quaternized polysulfone with reduced graphene oxide for highly stable anion exchange membranes. J. Membrane Sci..

[48] Lan R., Tao S., Irvine J.T. (2010). A direct urea fuel cell–power from fertiliser and waste. Energy Environ. Sci..

[49] Xu W., Zhang H., Li G., Wu Z. (2014). Nickel-cobalt bimetallic anode catalysts for direct urea fuel cell. Sci. Rep..

